# Handcuffs for bacteria - NDP52 orchestrates xenophagy of intracellular *Salmonella*

**DOI:** 10.15698/mic2015.06.208

**Published:** 2015-05-21

**Authors:** Pauline Verlhac, Christophe Viret, Mathias Faure

**Affiliations:** 1 CIRI, International Center for Infectiology Research, Université de Lyon, 69007 Lyon, France.; 2 Inserm, U1111, 69007 Lyon, France.; 3 CNRS, UMR5308, 69007 Lyon, France.; 4 Ecole Normale Supérieure de Lyon, 69007 Lyon, France.; 5 Université Lyon 1, Centre International de Recherche en Infectiologie, 69007 Lyon, France.

**Keywords:** autophagy, xenophagy, NDP52, bacteria

Macroautophagy (hereafter referred to as autophagy) is a catabolic process, which allows eukaryotic cells to degrade and recycle many deleterious intracellular components by capturing them in a vesicle, the autophagosome. As the keeper of cellular homeostasis, autophagy is highly regulated and can be modulated by either external or internal stimuli such as lack of environmental nutrients or intracellular pathogens, respectively. Dysregulation of the autophagic process has often been associated with human pathologies, ranging from neurodegenerative to chronic inflammatory diseases, which highlights the need for a better understanding of the process. During the past decade, our understanding of the molecular mechanisms of autophagy and its involvement in the clearance of intracellular pathogens has greatly improved. Basically, autophagy starts with the formation of a curved membrane, the phagophore. This membrane is then elongated until fusion of both extremities to generate a double membrane vesicle called the autophagosome, which entraps cytosolic components. Finally, the autophagosome fuses with a lysosome to form a degradative autolysosome; this process can be preceded by the fusion of the autophagosome with an endosome, forming a so-called amphisome.

During the elongation process, the growing phagophore can selectively capture cytosolic cargo. This selectivity is ensured by the autophagy receptors that recognise elements to be degraded on the one hand and a member of the ATG8 family (LC3s or GABARAPs proteins) bound to the phagophore on the other hand, via a specific LC3-interacting Region (LIR) motif. Cargo may either consist of cellular components, such as protein aggregates or defective organelles, or pathogens in the course of an infection. NDP52 is one of the autophagy receptors engaged during *Salmonella* Typhimurium infection: it can recognise either GALECTINE8 bound to damaged vacuoles that contain *S.* Typhimurium or ubiquitin decorating cytosolic bacteria directly. Recently, it was shown that NDP52 binds to LC3C through a non-canonical LC3-interacting region (CLIR), what is crucial for the targeting of *S. *Typhimurium to the phagophore. However, the mechanisms driving autophagosome maturation for the ultimate degradation of cargo remain to be fully understood, especially in the context of infections.

Interestingly, the endosomal membrane protein TOM1 and dynein motor MYOSIN VI were recently shown to be implicated in endosome trafficking and subsequent autophagosome maturation in healthy cells. Moreover, the absence of all three autophagy receptors, meaning OPTINEURIN, T6BP and NDP52, seemingly triggered a defect in both autophagosome biogenesis and maturation. As NDP52 was reported to contain a MYOSIN VI binding domain, we hypothesised that NDP52 could also be involved in autophagosome maturation. We could corroborate this hypothesis, as we observed that the single absence of NDP52 in healthy cells resulted in immature autophagosome accumulation. We have shown that NDP52’s MYOSIN VI binding domain as well as a newly identified LIR-like motif, which mediates its interaction with LC3A, LC3B or GABARAPL2 (but not LC3C) are both essential to perform NDP52-mediated autophagosome maturation. The LIR-like motif differs slightly from the canonical LIR motif by the absence of a hydrophobic residue in position X_3_. Importantly, we found that in human cells infected with *S*. Typhimurium, the MYOSIN VI binding domain and the LIR-like motif of NDP52 were both necessary to control the infection. By contrast, none of the interactions mediated by these domains were required to target bacteria to the autophagy machinery, which was exclusively restricted to the CLIR motif of NDP52.

Having the same protein addressing the pathogen to the autophagy machinery and ensuring its degradation could be an important evolutionary advantage against infections. This efficiency could help to reduce the delay necessary for maturation, thus avoiding escape of the pathogen from the autophagosome, or its adaptation to its new environment, which could lead to the establishment of a replicative niche. Of note, the conservation of both the CLIR and the LIR-Like motif of NDP52 among several primate species could suggest such a selective pressure. Indeed, beyond primates and among common animal models only ferrets seem to exhibit both CLIR and LIR-Like domains on NDP52 (see Table 1). Such differences could account for diverse responses of NDP52 towards stress or infection among animal species as already demonstrated for Chikungunya virus. Indeed, during Chikungunya virus infection human NDP52 plays a pro-viral role while murine NDP52 does not play such a role. Thus, pathogens could easily counteract xenophagy at several steps by manipulating NDP52, or any other autophagy receptors, which would play similar roles; functional redundancy among autophagy receptors could however ensure a selective immune advantage against pathogens targeting one of these receptors. For instance, the mechanism we described could help reducing cellular collateral damage by focusing autophagic degradation exclusively on invading pathogens while improving antigen processing in antigen-presenting immune cells.

**Figure 1:**
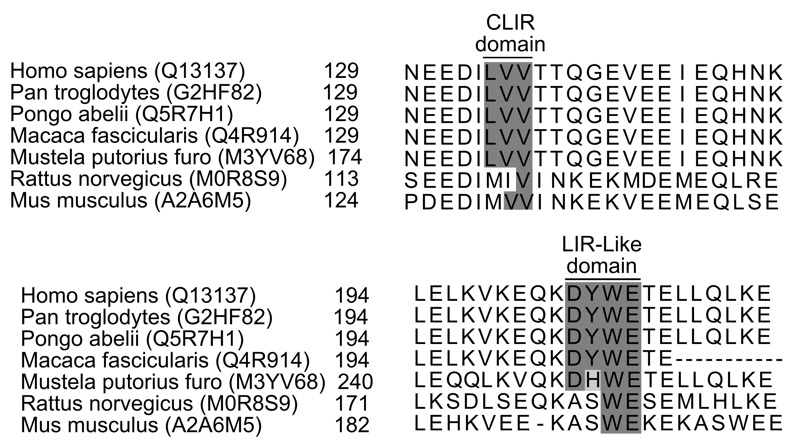
Figure 1: Alignment of NDP52 region encompassing the so-called CLIR motif (required for bacteria targeting to autophagosomes), and LIR-like motif (required for bacteria-containing autophagosome maturation).

Our study sheds light on the mechanisms underlying autophagosome maturation in both infected and healthy cells. NDP52 appears as a crucial actor during xenophagy as it both addresses the pathogen to the phagophore and regulates subsequent autophagosome maturation thus ensuring proper degradation of invading pathogens. Among others, two important questions arise from this work: whether other autophagy receptors share the same double function and how this mechanism is coordinated with the fusion process.

